# Enhancement of the Green H_2_ Production by Using TiO_2_ Composite Polybenzimidazole Membranes

**DOI:** 10.3390/nano12172920

**Published:** 2022-08-24

**Authors:** Sergio Díaz-Abad, Manuel A. Rodrigo, Cristina Sáez, Justo Lobato

**Affiliations:** Chemical Engineering Department, Enrique Costa Building, University of Castilla-La Mancha, Av. Camilo Jose Cela n 12, 13071 Ciudad Real, Spain

**Keywords:** SO_2_ depolarized electrolysis, green hydrogen, high-temperature electrolysis, composite membrane, TiO_2_, polybenzimidazole, SO_2_ crossover, proton conductivity, chemical stability

## Abstract

This study reports the hydrogen production using TiO_2_ based composite polybenzimidazole membranes through the SO_2_ depolarized electrolysis that requires lower energy input than the direct water electrolysis. Composite membranes prepared and studied in this work showed very promising results in terms of proton conductivity, chemical stability, and crossover. Thus, a reduction in SO_2_ crossover was observed with the increase of the concentration of TiO_2_, obtaining reductions as high as 42% with the 3.0 wt% TiO_2_-PBI membrane at 120 °C. Higher hydrogen production rates and Faradaic efficiencies were achieved by all the composite membranes, with an optimum for the 1.0 wt% TiO_2_-PBI membrane (with this membrane, the production of hydrogen increased a 53% at 110 °C and a 49% at 120 °C as compared with the standard PBI membrane), demonstrated the benefit of the use of composite membranes with respect to the standard one for green hydrogen production.

## 1. Introduction

Renewable energies are of paramount importance for a clean and sustainable development of the energy sector. However, the transition from a carbon-based sector to a green energy sector has some steps that are of concern and challenges that have to be addressed. One of them is that renewable energy production is intermittent and very difficult to predict. Therefore, the energy sector cannot rely on them to meet the energy demand of the population. There can be availability problems of electricity produced by these renewable energies that would cause shortage [1]. Consequently, the development of energy storage technologies capable to store the excess of produced renewable energy and to release it when needed is key to address these challenges. A promising method to store these energies is to do it in the form of hydrogen as energy vector [2]. For green hydrogen production, water electrolysis is the benchmark. However, due to the high energy requirements for this process, new alternatives are being developed [3,4,5,6]. Among those alternatives, the Hybrid Sulfur (HyS) cycle is an increasingly popular process for this green hydrogen production [7]. The HyS cycle has three steps of which the main one is the electrolysis step, in which hydrogen is generated, which benefits of a very low E^0^ = 0.158 V compared to the E^0^ = 1.210 V of water electrolysis. Therefore, the first of the three steps is the electrolysis (sulfur dioxide depolarized electrolysis, SDE) of water and sulfur dioxide (Equation (1)), which produce protons that recombine into hydrogen (Equation (2)) in the cathode of the electrolyzer and sulfuric acid which, in the second step, is thermally decomposed into water, sulfur dioxide and oxygen (Equation (3)). Finally, in the third step, oxygen and sulfur dioxide are separated, and then SO_2_ is recirculated to the electrolysis step.

(1) $${\;\mathrm{SO}}_{2}{+2H}_{2}O\;\to H_{2}{\mathrm{SO}}_{4}{+2H}^{+}{+2e}^{-}$$

(2) $${\;2H}^{+}{+2e}^{-}\;\to H_{2}$$

(3) $$H_{2}{\mathrm{SO}}_{4}\;\to {\mathrm{SO}}_{2}{+H}_{2}O+\frac{1}{2}{\;O}_{2}$$

This electrochemical step, where hydrogen is produced, is normally carried out in a proton exchange membrane (PEM) reactor in which the core of the configuration is the membrane electrode assembly (MEA). The MEA has two components of equal importance, the membrane and the catalyst layer. The catalyst layer has been the most studied part, for which noble catalysts such as platinum, palladium or gold have been tested, as well as non-noble catalysts and bi-metallic catalysts [8,9,10,11,12,13]. However, little attention has been paid to the membrane. Most works have employed Nafion membranes at temperatures lower than 80 °C, as it is an operational limit for these kind of membranes [14,15,16]. On the other hand, some works recommend the electrolysis to be above 100 °C for a better water management and to increase the overall thermal efficiency of the HyS cycle [17,18,19]. Nevertheless, there are only a few works that study the electrolysis step at high temperature. In this regard, Nafion must be replaced by a more suitable material. PBI is an excellent alternative to the Nafion membrane because of its excellent properties as membrane material at high temperature for electrochemical applications. On this matter, Jayakumar et al. [20] tested PBI and Nafion membranes for the SDE at 80 °C, obtaining better sulfuric acid production for the PBI membrane. Peach et al. [21] also studied PBI membranes up to temperatures of 120 °C, reporting a considerable increase in performance when the temperature increased from 80 °C to 120 °C. Díaz-Abad et al. [22] studied the influence of the temperature up to values of 170 °C with PBI membranes doped with H_3_PO_4_. On the other hand, further enhancement of the membranes has to be done in terms of durability under high acidic conditions and reducing the crossover of SO_2_ which will lead to the production of catalysts poisons such as sulfur or hydrogen sulfide. Adding fillers to the membrane can increase mechanical properties, lead to acid uptake due to the interaction between the filler and the doping acid, and it can also act as barrier to prevent the crossover of undesired species [23]. The addition of fillers has already been proven in PEM fuel cells (PEMFC) [24,25,26,27].

Furthermore, in a previous work, we employed an organic filler to prepare composite Graphene oxide/PBI membranes which were tested up to 140 °C, showing a superior performance when the GO content was 2.0 wt% in terms of reached current density and hydrogen production [28,29]. In this regard, inorganic fillers have also proven to be a good candidate to be used in proton exchange membranes at high temperature. Titanium dioxide is such an inorganic filler, which has already been used into PBI membranes for fuel cell applications [30,31,32,33], demonstrating its benefits in reducing gas crossover and increasing the acid retention and the acid doping level. Therefore, in this work, TiO_2_-PBI membranes with different TiO_2_ concentrations will be evaluated for the sulfur dioxide depolarized electrolysis, aiming to reduce the crossover of SO_2_ from the anode of the cell which, in turn, will help to obtain hydrogen with higher purity. In other works, very little attention has been paid to the actual production of hydrogen, and membranes have only been evaluated in terms of reached current density values. However, in this work, actual hydrogen was measured and used as a key parameter to evaluate the effect of TiO_2_ into the PBI matrix.

## 2. Experimental

### 2.1. Materials

Commercial PBI solution was obtained from PBI Performance Products (Charlotte, NC, USA) with a PBI concentration of 26 wt% with N,N-dimethylacetamide (DMAc) as solvent and stabilized with LiCl. DMAc was purchased from Panreac (Barcelona, Spain) and used with no further purification. Titanium dioxide (TiO_2_) particles (99.9%) were received from Alfa Aesar (Kandel, Germany) with a particle size of 32 nm. Phosphoric acid was obtained from Merck (Darmstad, Germany).

### 2.2. Membrane Preparation 

The composite membranes containing TiO_2_ were prepared with contents of TiO_2_ from 0.5% to 3% (0.5, 1.0, 2.0, and 3.0 wt%) by the solvent casting method as described elsewhere [28]. Briefly, TiO_2_ nanoparticles were dispersed in DMAc for 15 min in an ultrasound bath. Then, in a different vial, a diluted PBI solution was prepared by adding DMAc to the commercial solution. Once a homogeneous PBI solution was obtained, both solutions were mixed, obtaining a final PBI concentration of 2.0 wt% for membrane casting. This solution was further homogenized in the ultrasound bath for 2 h. The films were obtained by pouring the prepared solutions in a glass plate (13 cm diameter) and evaporating the solvent at 80 °C in an oven for 24 h. The final membranes were detached form the plate using DI water [34,35]. Prior to doping the membranes for further characterization and for the electrolysis experiments, they were washed with boiling water during 2 h and then dried again at 80 °C. 

### 2.3. Chemical and Physicochemical Characterization

#### 2.3.1. XRD & SEM

XRD spectra for the pristine and composite membranes was obtained by X-Ray Diffraction in a Philips X’Pert MPD (PANALYTICAL, Malvern, UK) diffractometer applying Kα corresponding to the transition from copper radiation (λ = 1.5404 Å). Data were obtained recording the 2θ angular region from 10° to 100° (scan rate 0.02°·s^−1^), and 2 cm^2^ samples were used. 

The SEM pictures were carried out in a Microscope Gemini SEM 500 (Oberkochen, Germany) field emission. To prepare the samples, all the membranes were sputter-coated with a very thin layer of gold. The thickness of this conductor layer was 2 nm.

#### 2.3.2. Ionic Conductivity

The conductivity of the membranes was measured using the four-points system. The system is formed by a stainless-steel plate (0.1 cm of thickness) with four perforations where gold wires are placed. The plate was electrically insulated using a Teflon sheet (W.L. Gore and Associates, Newark, DE, USA). The sample (6.0 × 1.0 cm) is placed on the top of the plate and the wires are placed on the top of the membrane which are connected to a galvanostat/potentiostat (AutoLab PGSTAT204, Barendrecht, The Netherlands) equipped with a frequency response analysis module (FRA32 module). The device was thermally insulated from the exterior while performing the measurements and connected to a heater. After 1 h of reaching the desired temperature, electrochemical impedance spectroscopy (EIS) analysis was carried out (frequency range 100–10,000 Hz and amplitude of 10 mV). Proton conductivity was calculated using Equation (4). R_Ω_ (ohm) represents the obtained value of the resistance to the ionic flux, S (cm^2^) is the transversal section of the sample, and l (cm) is the distance between the wires where a potential difference is measured.

(4) $$\sigma \;\left(\frac{S}{\mathrm{cm}}\right)=\frac{1}{R_{Ω}}*\frac{l}{S}\;$$

#### 2.3.3. Acid Doping Level (ADL)

Samples of 2.0 × 2.0 cm^2^ were soaked in 85 wt%H_3_PO_4_. The moisture of the membranes was first removed by drying them at 80 °C overnight (m_dry PBI_). Then, the membranes were weighted and doped in phosphoric acid for 24 h at 60 °C. When the wet weight of the doped PBI membranes was constant, the samples were dried again to remove the absorbed water. Then, the membranes were weighed (m_doped PBI_) again to calculate the acid uptake using Equation (5).

(5) $${\mathrm{ADL}\;[\mathrm{mol}\;H}_{3}{\mathrm{PO}}_{4}\cdot r.u{.\mathrm{PBI}}^{-1}]=\frac{\left(m_{\mathrm{doped}\;\mathrm{PBI}}{-m}_{\mathrm{dry}\;\mathrm{PBI}}\right)*98\frac{g}{{\mathrm{mol}}_{H_{3}{\mathrm{PO}}_{4}}}}{\frac{m_{\mathrm{dry}\;\mathrm{PBI}}}{308\frac{g}{r.u.\;\mathrm{PBI}}}}$$

#### 2.3.4. Phosphoric Acid Retention Capability

Acid retention of the prepared membranes was measured as follows. Doped samples (2.0 × 2.0 cm^2^) were immersed in a glass bottle with deionized water at 80 °C for one hour while magnetically stirred. The concertation of phosphates was monitored during the experiment by taking liquid samples each 10 min, which were measured by ionic chromatography.

#### 2.3.5. Contact Angle Measurement

Water management is a very important parameter for water splitting processes. Therefore, the contact angle of a drop of water was used to determine the affinity of the membranes towards water. To do so, a water drop is dropped on top of the sample and a picture is taken after 15 min of stabilization. Then, the right and left contact angles are determined from the interface of the water drop and the membrane.

#### 2.3.6. Chemical Stability

Chemical resistance, of the nanocomposite membranes and the pristine PBI membrane, was measured by performing an accelerated oxidation test with the sulfate radical as oxidizing agent (SO_4_^−^·). The selection of this radical (and not OH·) was due to the very high oxidation power of this radical [36] which is more likely to be formed in the sulfur based electrochemical reactor due to side reactions at high [37] in the acidic sulfur environment. For the persulfate test, dried PBI based membranes (45 mg) were introduced in a flask with an initial concentration of Na_2_S_2_O_8_ (Sigma Aldrich, Burlington, MA, USA) of 500 ppm in H_2_SO_4_ 1.0 M. The experiments were carried out at 80 °C, until membrane failure was observed.

(6) $$S_{2}O_{8}^{2-}\overset{\mathrm{Heat}}{\to }{\;2\;\mathrm{SO}}_{4}^{-}\cdot \;$$

### 2.4. SO_2_ Crossover Measurement

Sulfur dioxide crossover was measured by means of electrochemistry as follows. MEASs were prepared with a catalyst loading of 0.7 mgPt·cm^−2^ in both anode an cathode with an membrane area of 4 cm^2^. The working electrode was fed with a mixture of steam and nitrogen. The counter electrode was first fed with nitrogen and a constant voltage of 0.7 V was applied to create a baseline and once it was obtained, SO_2_ (70 mL·min^−1^. Then, the new current value obtained is recorded until reaching a constant value. The crossover was calculated as the gap between the baseline and the current when flowing sulfur dioxide that crossed the membrane and underwent oxidation according to Equation (1). This process was repeated for all the studied temperatures. Thus, the flux of SO_2_ which crosses the membrane is proportional to the net measured current. This procedure was also followed by Opperman et al. to measure the transport of SO_2_ through PBI membranes [38]. 

### 2.5. Sulfur Dioxide Depolarized Electroylsis

A 25 cm^2^ electrolyzer was employed for the electrochemical characterization for the pristine and composite membranes (Figure S1 depicts the experimental set-up). In order to evaluate the effect of temperature, the electrochemical cell was characterized in the temperature range of 110 °C and 140 °C. Prior the experiments, the membranes were doped in 85 wt% H_3_PO_4_ at 80 °C for 12 h. Both anode and cathode were prepared with a catalyst loading of 0.7 mgPt·cm^−2^ by air dispersion of an ink containing commercial Pt/C (40 wt% of Pt with Vulcan XR-72) with PBI as ionomer and DMAc as solvent. This ink was sprayed onto H23C2 GDL gas diffusion layers (Freudenberg, Weinheim, Germany). The electrolyzer was finally assembled with eight equidistant bolts and employing a compression toque of 1 N-m.

Electrolyzer operation was carried out as follows. Water flow was 0.2 mL min^−1^ in liquid phase (λ = 2) and SO_2_ gaseous flow was 70 mL·min^−1^ in the anode of the electrolyzer. Before entering the electrolyzer, water and SO_2_ were mixed in a “T” connection. Then, they pass through a stainless-steel tube connected to a heater (110 °C) to vaporize the water. On the other hand, a mixture of nitrogen (100 mL min^−1^) and steam (0.5 mL min^−1^ of liquid water) is fed. For each temperature, the anode was collected to measure the production of H_2_SO_4_ at 0.60 V by measuring the produced sulphates by ionic chromatography. The system was electrochemically characterized using of linear sweep voltammetries (LSVs) (0.1 to 1.0 V at a scan rate of 10 mV s^−1^) using a galvanostat/potentiostat (AutoLab PGSTAT204, Barendrecht, Netherlands). EIS measurements were carried out at 0.7 V with an amplitude of 20 mV and a frequency range of 0.1–10,000 Hz. A model R(RC), shown in Figure S2, was employed for the fitting of the experimental EIS data to obtain the ohmic and charge transfer resistance values. Hydrogen obtained in the cathode was characterized by gas chromatography with a GC-2030 (Shimadzu, Japan) equipped with a Rxi-1ms column (L = 30 m; ID = 0.32 mm; DF = 0.50 µm) for sulfurous compounds (H_2_S and SO_2_) and a Rt-Msieve 5A column (L = 30 m; ID = 0.32 mm; DF = 30 µm) for the identification of small gas molecules (H_2_, N_2_ and O_2_).

## 3. Results and Discussion

**Figure 1:**
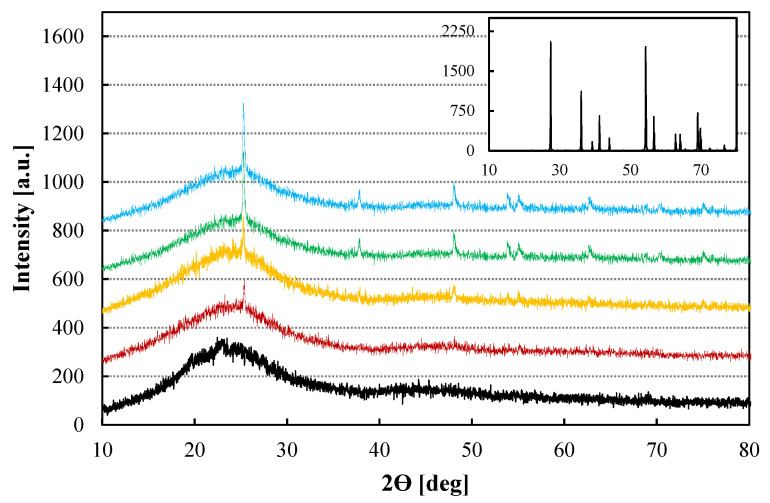
XRD data for the pristine and TiO_2_-PBI composite membranes. Inset: XRD of pure TiO_2_ employed in this work. Black line: Std-PBI; Red line: 0.5 wt% TiO_2_-PBI; Yellow line: 1.0 wt% TiO_2_-PBI; Green line: 2.0 wt% TiO_2_-PBI; Blue line: 3.0 wt% TiO_2_-PBI.

Figure 1 shows the XRD data of the PBI composite membranes with different TiO_2_ contents and in the inset the XRD pattern of the TiO_2_ used as filler. It can be observed that the main peaks of the TiO_2_ compound corresponds with the crystalline phase of Rutile as the mean peaks of rutile phase at 2Θ =27.5°, 54.3°, and 36.1° appear [39]. On the other hand, the typical broad peak at 2Θ =25°, which results from the convolution of amorphous and crystalline scattering, is observed [31]. Regarding the composite PBI based membranes, it can be clearly distinguished that the higher the content of rutile was, the more intense was the main peak. Furthermore, the other peaks of the TiO_2_ rutile phase appeared more clearly in the plot when the content of TiO_2_ was 2.0 wt% or higher. These results confirm that the inorganic filler is within the polymer matrix and the structure of it has not changed when it was introduced in the polymeric film.

**Figure 2:**
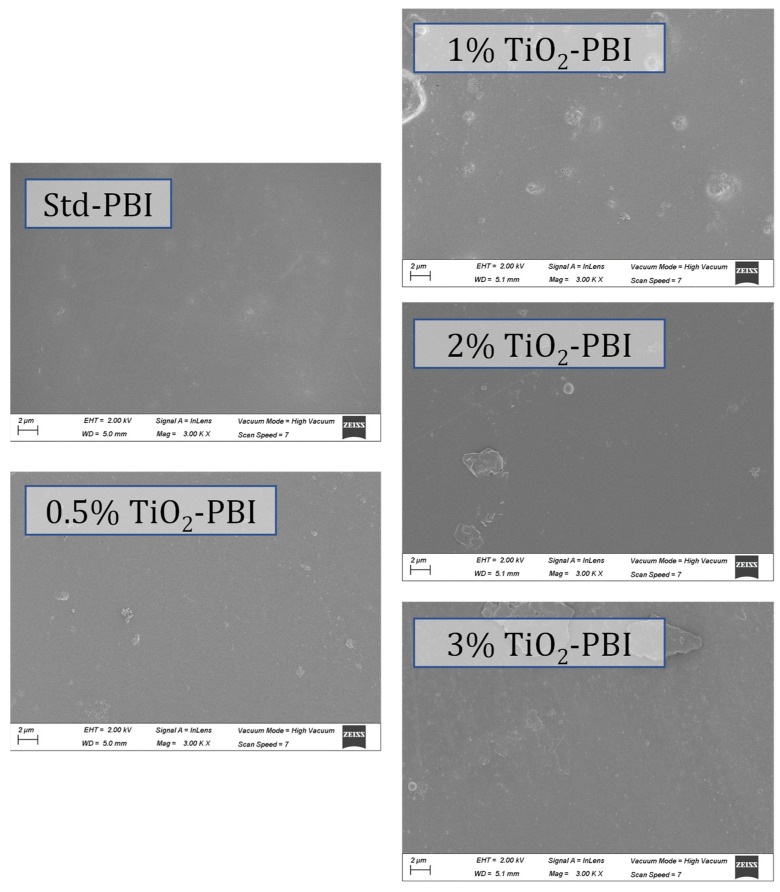
Surface SEM images for the standard and composite membranes.

On the other hand, SEM surface characterization (Figure 2) shows a good interaction between the TiO_2_ nanoparticles and PBI. As observed, the surfaces do not show imperfections apart from the apparition of TiO_2_ particles covered by the polymer, which indicates good compatibility between PBI and the inorganic filler.

Two key parameters for PEM electrolyzers are the water management and the chemical stability of the membranes. Regarding water management, the contact angle of the membranes was measured (Figure S3). When a small amount of TiO_2_ is added to the PBI matrix, the behaviour of the membrane changes from hydrophilic to an almost hydrophobic performance. This can be attributed to changes in the surface of the membrane associated to the introduced particles. In addition, the similar values for the right and left contact angle indicate a homogeneous surface of the membranes. Chemical stability of the prepared membranes was evaluated by means of sulfate radicals attack (Figure S4), which are expected to be formed in a sulfur environment. There is a great improvement when adding TiO_2_ as the time at which the membrane broke increased from 8 h for the pristine PBI membrane to 30 h for the 0.5 wt% TiO_2_-PBI membrane, 40 h for the 1.0 wt% TiO_2_-PBI membrane, 42 h for the 2.0 wt% TiO_2_-PBI membrane and 42 h for the 3.0 wt% TiO_2_-PBI membrane. Thus, adding a filler created a more compact structure capable of protecting the polymer chains from the attack of the sulfate radicals. These results are in line with the addition of other fillers to the PBI matrix which also increase it chemical stability [28].

The doping level and acid retention capability are two main features of phosphoric acid doped PBI based membranes to be evaluated for SO_2_ depolarized electrolysis for hydrogen production as the first one is related to the conductivity and the other to the durability issues of the system. In Table 1, it can be seen that these two parameters and the thickness, as well, increase when the membrane is doped for all the membranes studied in this work. At a first sight, it could be said that there is not a clear tendency regarding the doping level with the content of TiO_2_. Nevertheless, it can be stated that these results agree with previous results when different contents (2.0 wt%, 4.0 wt%, 8.0 wt% and 16.0 wt%) of TiO_2_ were added to a PBI membrane for a HT-PEMFC [30]. In that study, the optimum filler content was 2.0 wt% as higher values of TiO_2_ led to doping levels even lower than those attained in the pristine PBI membrane. It has been found in the literature, that inorganic oxides tend toward the agglomeration in the polymeric solution and, after solvent evaporation step, the active surface for acid absorption diminished [40,41]. In this work, the range of TiO_2_ content is narrower as we knew those results and, in this case, it seems that small amounts of TiO_2_ as filler are adequate to improve the doping level with respect to the standard PBI membrane, being 0.5 wt% the one with the highest doping level and, hence, the one with the highest thickness increase (as this parameter is closely related with the doping level). Thus, for the case of the best membrane, from the doping point of view (the one with 0.5 wt%), the doping level increased 11.4% and the thickness 13.8% with respect to the standard PBI.

Acid leaching tests for all the membranes were performed under the same operation conditions to assess the acid retention capacity, which is considered one of the main causes of a loss of conductivity for fuel cell technology and, hence, for electrolysis applications [42,43,44]. It can be observed in Table 1, the positive effect of the presence of TiO_2_ as filler, regardless of the content, at least, the acid retention capacity doubled with respect to the pristine PBI membrane. Furthermore, the acid retention capability increases more than three times when the content of titania is 1.0 wt%, and this effect is achieved also by the membranes with 2.0 and 3.0 wt%. The effect observed agrees with our previous work where TiO_2_ and TiOSO_4_ were used as fillers in PBI based membranes [45], and with results obtained by Mustarelli et al. [43] where it was found that the addition of 5.0 wt% of silica in a PBI matrix reduced the acid leaching from the membrane by more than a factor of two.

**Figure 3:**
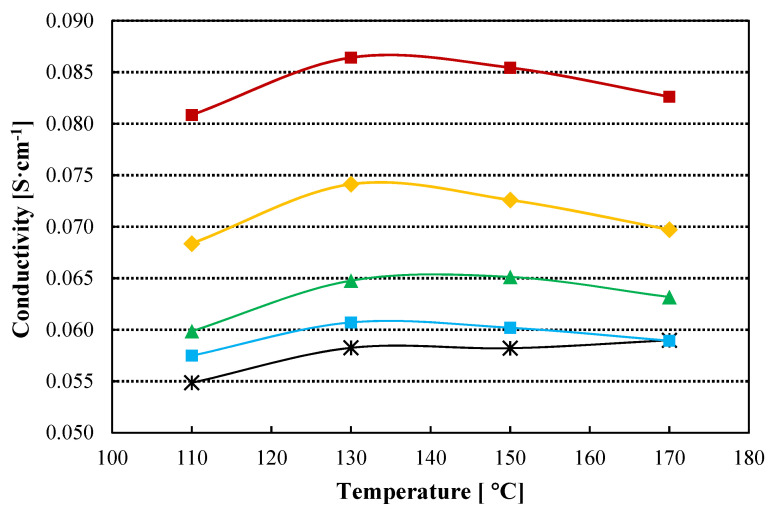
In plane conductivity for the prepared membranes. Doped with 85 wt% H_3_PO_4_. Black line: Std-PBI; Red line: 0.5 wt% TiO_2_-PBI; Yellow line: 1.0 wt% TiO_2_-PBI; Green line: 2.0 wt% TiO_2_-PBI; Blue line: 3.0 wt% TiO_2_-PBI.

Proton conductivity is one of the most useful parameters to anticipate the behaviour of the membrane in an electrolyzer. In-plane conductivity of the standard and the composite TiO_2_-PBI doped with phosphoric acid is shown in Figure 3. It can be observed, that the in-plane conductivity is higher for all the composite membranes compared with the pristine PBI membrane, with a maximum in conductivity for all the samples at 130 °C. Temperature does not play a significant role in this case, because there are not significant changes when increasing temperature in the studied range. On the other hand, the slight decrease in conductivity when temperature is higher than 130 °C can be related to phosphoric acid demineralization associated to losses of water, as has been pointed out by Ma et al. [46]. On the other hand, a considerable increase is obtained for the composite membrane with a TiO_2_ content of 0.5 wt% with a value of 0.086 S cm^−1^ at 130 °C as compared with the conductivity of 0.058 S cm^−1^, measured for the standard PBI membrane. Then, the conductivity of the composite membranes decreases when the amount of the filler increases. This behaviour can be correlated with the acid doping level (Table 1). The increase in acid doping level with a maximum for the 0.5 wt% TiO_2_ membrane explains its conductivity above the other membranes, then the in-plane conductivity decreases with the decrease in the ADL. Nevertheless, the conductivity for the 1.0 wt% TiO_2_ composite membrane is 28% higher than for the pristine membrane (0.074 S cm^−1^), 12% higher for the 2.0 wt% composite membrane (0.065 S cm^−1^), and 5.0% higher for the 3.0 wt% TiO_2_ membrane.

**Figure 4:**
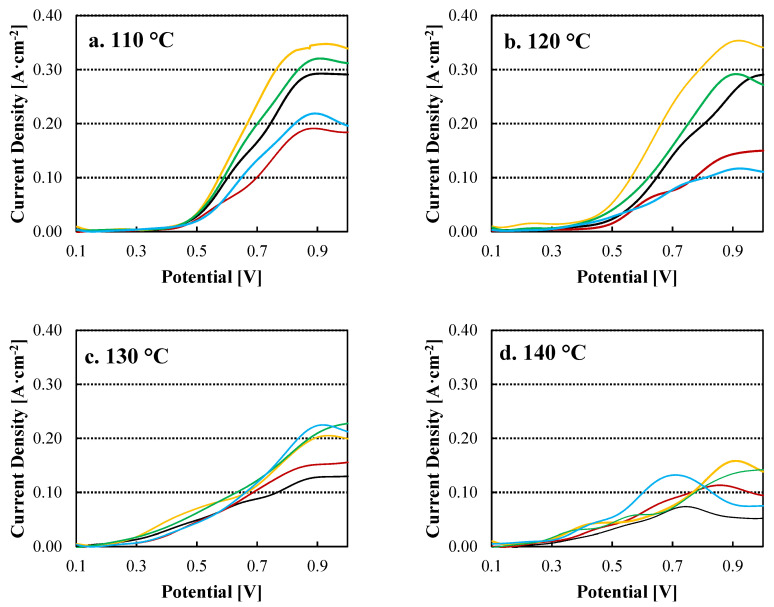
Linear sweep voltammetries for the pristine and composite membranes at different temperatures. Black line: Std-PBI; Red line: 0.5 wt% TiO_2_-PBI; Yellow line: 1.0 wt% TiO_2_-PBI; Green line: 2.0 wt% TiO_2_-PBI; Blue line: 3.0 wt% TiO_2_-PBI. (**a**) 110 °C; (**b**) 120 °C; (**c**) 130 °C; (**d**) 140 °C.

The electrolysis performance for the prepared membranes is shown in Figure 4. The addition of TiO_2_ to the PBI matrix clearly benefits the electrolyzer performance at 110 °C and 120 °C for a filler concentration of 1.0 wt%. For example, a current density of 0.13 A cm^−2^ at 0.6 V and 0.34 A cm^−2^ at 0.85 V was obtained for the 1.0 wt% TiO_2_-PBI membrane which was a value 44% higher at 0.6 V and 21% higher at 0.85 V than for the pristine PBI membrane (0.09 A cm^−2^ and 0.28 A cm^−2^ respectively). Then, at 120 °C, there is a slight improvement at a potential of 0.6 V as the 1.0 wt% membrane reaches a current density of 0.15 A cm^−2^, demonstrating that temperature enhances the kinetics in the electrolyzer. However, for the standard membrane, a current density of 0.07 A cm^−2^ at 0.6 V is obtained, increasing the improvement of a TiO_2_ content of 1.0 wt% to 114% in terms of current density compared to the pristine PBI membrane. Increasing the filler content to a 2.0 wt% also improves the results of the pristine PBI membrane, however, this membrane showed current density values lower than the 1.0 wt% TiO_2_-PBI membrane. This can be attributed to the in-plane conductivity, which is lower when increasing the filler content. On the other hand, for the minimum and maximum TiO_2_ contents, the obtained results worsen those obtained for the pristine membrane. In the case of the 0.5 wt% TiO_2_ membrane, the high conductivity obtained can be rapidly lost due to the lower acid retention of this membrane and, also, the low content of TiO_2_ in this membrane might not be enough to see any improvement in the electrolyzer. Then, the membrane with the highest content of TiO_2_ presented a very similar conductivity as compared with the standard membrane. Moreover, a high filler content can disrupt proton transport channels through the membrane, decreasing electrolyzer performance [45]. At 130 °C and 140 °C, there is a noticeable loss of performance in the electrolyzer. Figure 4c,d show almost no difference in the polarization curves when the electrolyzers are equipped with the pristine PBI membrane or any of the TiO_2_ composite membranes. This effect of the temperature is attributed to side reactions that can happen in the HyS process due to SO_2_ crossover [47,48,49]. These results exhibit that side reactions (Equations (7)–(9)) are favoured at temperatures higher than 130 °C. Therefore, the catalyst can be poisoned by the formation of elemental sulfur (Equations (7) and (9)) or by the formation of hydrogen sulfide (Equation (8)).

(7) $${\mathrm{SO}}_{2}{+4\;e}^{-}{+4\;H}^{+}\;\to {\;S+2\;H}_{2}O\;$$

(8) $${\mathrm{SO}}_{2}{+3\;H}_{2}\;\to {\;H}_{2}{S+2\;H}_{2}O\;$$

(9) $${\mathrm{SO}}_{2}{+2\;H}_{2}S\;\to {\;3\;S+2\;H}_{2}O\;$$

Considering the obtained results, the depolarized electrolysis of SO_2_ is not the only sulfur-based reaction taking place in the reactor and a deep product characterization should be carried out to observe the formation of those un-desired products.

The drastic change in the behaviour of the electrolyzer when increasing temperature is also observed in the EIS data shown in Table 2 (Nyquist plots reported in Figure S5). One of the parameters that EIS allows to determine is the ohmic resistance of the reactor, with the membrane being the main contributor for this resistance. Variations in the ohmic resistance are attributed to the different composition of the membranes. The lowest value is obtained for the composite membrane 1.0 wt% TiO_2_-PBI, which already showed a high in-plane conductivity and a high acid retention. The higher value in the resistance measured for the membrane with a TiO_2_ content of 0.5 wt% can be explained by a low acid retention which, in turn, causes an increase in resistance over the course of the experiment. It can be observed that temperature does not play a relevant role for the ohmic resistance, in fact, the formation of elemental sulfur (Equations (7) and (9)) could minimize the beneficial effect of temperature [14,28]. Regarding the charge transfer resistance, the obtained results help to understand the trend of the linear sweep voltammetries reported in Figure 4. When adding TiO_2_ to the PBI matrix, a positive effect is observed in all the range of temperature. However, only for contents of 1.0 wt% and 2.0 wt% this improvement is of relevance as for the other two composite membranes the charge transfer resistance is very similar to the obtained for the pristine PBI membrane. The lower acid doping level of the 3.0 wt% TiO_2_-PBI membrane and its very similar conductivity to the pristine PBI membrane explains the results shown in Table 3. Then, as aforementioned, the low performance of the 0.5 wt% TiO_2_-PBi membrane is attributed to its low phosphoric acid retention. When increasing temperature from 120 °C to 130 °C, the charge transfer resistance drastically increases, demonstrating that side reactions are being favoured at those temperatures.

**Figure 5:**
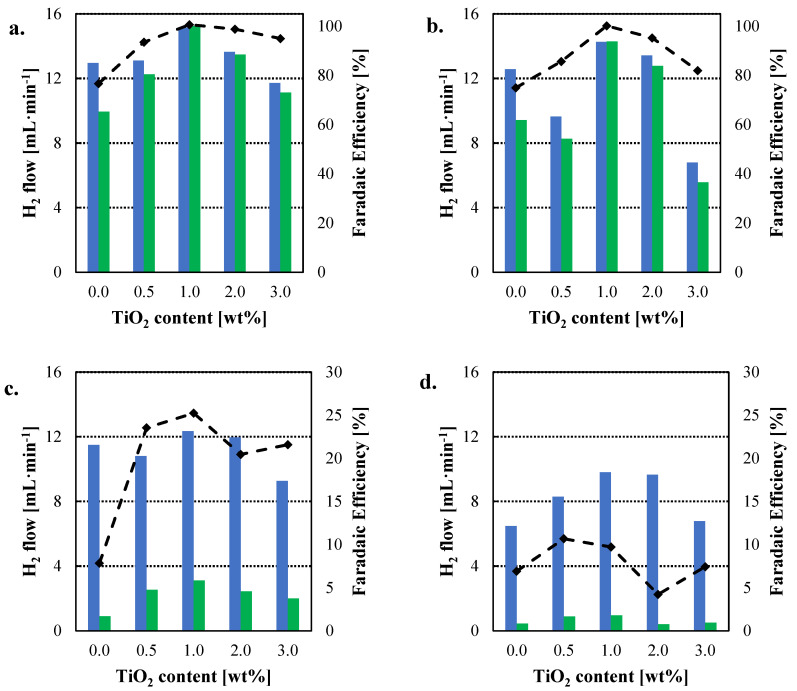
Hydrogen production rate for the pristine and composite membranes; (**a**). 110 °C; (**b**). 120 °C; (**c**).130 °C; (**d**). 140 °C. Blue columns: theoretical faradaic hydrogen rate; Green columns: experimental hydrogen production rates; Dashed line: Faradaic efficiency.

Even though obtaining high current densities is of importance for the operation of the electrolyzer, as it gives an idea of the reaction rate, the most essential parameter is the production of hydrogen, as its obtention is the purpose of the water molecule splitting processes. However, this production of hydrogen has never been reported for other HyS works, except for our previous work [28]. Figure 5 shows the actual production of hydrogen as function of temperature and the Faradaic efficiency for each of the employed membranes. Faradaic efficiency was calculated using Equation (10), where *I* is the intensity recorded at 0.6 V, *t* is the unit of time, *F* is faraday’s constant (96,500 C mol^−1^), *n* the number of electrons (2), and $\rho_{H_{2}}$ is the hydrogen density (0.041 mol L^−1^) at 20 °C and 1 bar.

(10) $$\mathrm{Faradaic}\;\mathrm{efficiency}\;\left[\%\right]=\frac{{\mathrm{Actual}\;H}_{2}\;\mathrm{flow}\;\left({L\cdot s}^{-1}\right)}{\left(\frac{I\;\left(A\right)\text{·}t\;\left(s\right)}{F\;\left({C\;\mathrm{mol}}^{-1}\right)\cdot n\;\left(e^{-}\right)}\right){\text{·}\rho }_{H_{2}}^{-1}\left({\mathrm{mol}\;L}^{-1}\right)}\cdot 100\;$$

The benefit of the incorporation of TiO_2_ as inorganic filler is clearly observed at 110 °C, obtaining higher hydrogen production rates and Faradaic efficiencies for the composite membranes, with an optimum for the 1.0 wt% TiO_2_-PBI membrane. For instance, 15.23 mL min^−1^ of hydrogen are obtained when the electrolyzer is equipped with the 1.0 wt% TiO_2_-PBI membrane and 13.48 mL min^−1^ for the 2.0 wt% TiO_2_-PBI compared to 9.93 mL min^−1^ for the pristine membrane. At a higher temperature of 120 °C, hydrogen production and Faradaic efficiency follow the same trend, with higher hydrogen production values for the membranes with a filler content of 1.0 wt% and 2.0 wt%, demonstrating the benefits of TiO_2_ as filler for PBI membranes for the HyS cycle. On the other hand, as previously observed in the polarization curves (Figure 4), there is a drastic drop at 130 °C and 140 °C. The hydrogen production rate is much lower than for the previous temperatures and the efficiency towards hydrogen production is also lower. Again, these results agree with the charge transfer resistance data, that prove the side reactions are occurring in the electrolyzer which are consuming the produced hydrogen to produce hydrogen sulfide (Equation (9)) or protons for Equation (8), where elemental sulfur is formed. This hypothesis is further confirmed by the levels of hydrogen sulfide measured from the outlet of the electrolyzer and showed in Figure S6. It can be observed that the production of HyS drastically increases at 130 °C when the production of hydrogen decays. At 140 °C, the formation of elemental sulfur is favoured over H_2_S (Figure S6), as the cathode outlet acquired a yellow colour at this temperature. That is the reason the molar ratio H_2_/H_2_S slightly increases at 140 °C. Furthermore, H_2_SO_4_ efficiency was measured by ionic chromatography (Figure S7). As for hydrogen, the efficiency towards the production of sulfuric acid drops at 130 °C for all the cases, demonstrating that the desired reaction is being limited.

Table 3 reports the molar ratio of H_2_ and H_2_S at each temperature for the pristine and the composite membranes. As observed, adding TiO_2_ as inorganic filler to PBI definitely benefits the electrolyzer performance towards the production of hydrogen. In this case, the composite membrane 1.0 wt% TiO_2_-PBI outperforms the pristine PBI membrane at 110 °C and 120 °C, and the other composite membranes also show very good results when compared with the pristine PBI membrane. By looking at Table 3, it can also be observed how the formation of hydrogen is inhibited by the formation of H_2_S when increasing temperature. 

**Figure 6:**
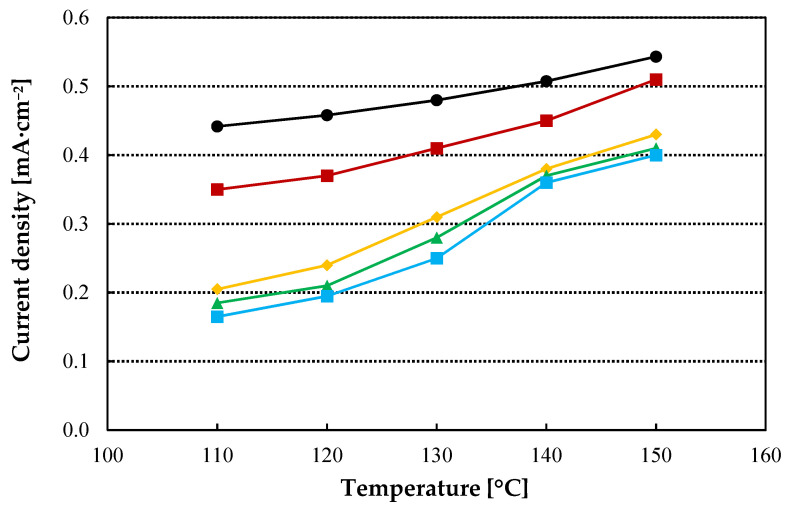
Crossover measurements for the pristine and TiO_2_-PBI composite membranes prepared in this work. Black line: Std-PBI; Red line: 0.5 wt% TiO_2_-PBI; Yellow line: 1.0 wt% TiO_2_-PBI; Green line: 2.0 wt% TiO_2_-PBI; Blue line: 3.0 wt% TiO_2_-PBI.

As aforementioned, the side reactions occurring in the cathode of the electrolyzer are caused by the crossover of sulfur dioxide. The improvement of the composite membranes can be, in part, attributed to a reduction in the crossover of sulfur dioxide. Figure 6 depicts the crossover of SO_2_ measured for the prepared membranes. Adding a small amount of 0.5 wt% of TiO_2_ already results in a clear reduction of the crossover. The crossover further decreases when increasing the content of the filler and reducing the measured current density due to the crossover of SO_2_ from 0.44 mA cm^−2^ to 0.15 mA cm^−2^ for the 3.0 wt% TiO_2_-PBI membrane. The addition of the fillers changes the inner structure of the membrane, reducing the free volume in the membrane and, also, acting as barriers for the transport of sulfur dioxide from the anode to the cathode of the cell. These crossover results help to comprehend the hydrogen production results and the best performance of the composite membranes compared to the pristine PBI membrane shown in Figure 4 and Figure 5. 

## 4. Conclusions

Composite PBI membranes containing TiO_2_ as inorganic filler have successfully been prepared for the electrolyzer of the HyS cycle. TiO_2_-PBI membranes demonstrated superior performance in terms of SO_2_ crossover reduction, proton conductivity, electrolysis performance, and more importantly, in terms of hydrogen production. This work shows the importance of measuring and characterizing the hydrogen outlet stream, in order to determine whether a change in any of the components of the electrolyzer improves its performance. In this regard, the composite membrane 1.0 wt% TiO_2_-PBI membrane showed the best results among the studied membranes. Regarding temperature, when this parameter is increased above 120 °C, there is a change in the kinetic rates of the system with an associated reduction in hydrogen production and Faradaic efficiency. Therefore, more efforts have to be made to enhance the materials for this, trying to prevent the crossover of SO_2_ to help increase temperature without losing hydrogen production efficiency.

## Figures and Tables

**Table 1:** ADL, thickness increase and acid retention results.

Membrane	Thickness Increase [%]	Doping Level [mol H_3_PO_4_ r.u. PBI^−1^]	Acid Retention [%]
Standard	106.7	11.4	22.5
0.5 wt% TiO_2_	121.5	12.7	51.8
1.0 wt% TiO_2_	119.7	12.2	73.5
2.0 wt% TiO_2_	108.2	11.8	71.2
3.0 wt% TiO_2_	105.8	10.9	69.8

**Table 2:** Ohmic resistance and charge transfer resistances for the studied membranes.

TiO_2_ [wt%]	110 °C		120 °C		130 °C		140 °C	
	R_Ω_ [mΩ]	R_P_ [mΩ]	R_Ω_ [mΩ]	R_P_ [mΩ]	R_Ω_ [mΩ]	R_P_ [mΩ]	R_Ω_ [mΩ]	R_P_ [mΩ]
0.0	15.8	42.2	16.0	57.2	15.5	180.0	18.3	322.0
0.5	16.1	50.5	17.6	55.4	19.3	129.0	19.2	157.0
1.0	10.2	24.1	11.5	25.3	11.6	72.4	12.1	129.0
2.0	12.8	28.4	13.7	42.5	14.1	79.4	14.3	135.0
3.0	13.5	45.8	14.6	81.4	15.4	156.0	16.3	207.0

**Table 3:** Ratio of the produced hydrogen and H_2_S in the cathode of the electrolyzer.

TiO_2_ [%]	Ratio H_2_-H_2_S at 110 °C	Ratio H_2_-H_2_S at 120 °C	Ratio H_2_-H_2_S at 130 °C	Ratio H_2_-H_2_S at 140 °C
0.0	57.12	11.94	0.30	1.06
0.5	228.39	16.22	0.93	2.19
1.0	303.23	70.78	1.59	5.94
2.0	262.23	45.82	1.17	4.41
3.0	173.74	24.11	0.91	3.89

## Data Availability

The data presented in this study are available on request from the corresponding author.

## Supplementary Materials

The following are available online at https://www.mdpi.com/article/10.3390/nano12172920/s1, Figure S1: Experimental Set-up, Figure S2: (RC) circuit use to fit the EIS data with the software NOVA, Figure S3: Contact angle for the different TiO_2_ concentrations, Figure S4: Chemical stability test. Time at which each membrane failed; Figure S5: Nyquist plots at 0.7 V for the studied membranes. Green dots: 110 °C; Blue dots: 120 °C; Orange dots: 130 °C; Red dots: 140 °C; Figure S6: H_2_S contents in the cathode outlet measured at 0.6 V; Figure S7: H_2_SO_4_ production efficiency in the anode measured at 0.6 V. Black column: Std-PBI; Red column: 0.5 wt% TiO_2_-PBI; Yellow column: 1.0 wt% TiO_2_-PBI; Green column: 2.0 wt% TiO_2_-PBI; Blue column: 3.0 wt% TiO_2_-PBI, Table S1: SO_2_ crossover flux.

## References

[1] Sun C., Negro E., Vezzù K., Pagot G., Cavinato G., Nale A., Herve Bang Y., Di Noto V. (2019). Hybrid Inorganic-Organic Proton-Conducting Membranes Based on SPEEK Doped with WO_3_ Nanoparticles for Application in Vanadium Redox Flow Batteries. Electrochim. Acta.

[2] Gahleitner G. (2013). Hydrogen from Renewable Electricity: An International Review of Power-to-Gas Pilot Plants for Stationary Applications. Int. J. Hydrogen Energy.

[3] Tong X., Ovtar S., Brodersen K., Hendriksen P.V., Chen M. (2019). A 4 × 4 Cm^2^ Nanoengineered Solid Oxide Electrolysis Cell for Efficient and Durable Hydrogen Production. ACS Appl. Mater. Interfaces.

[4] Caravaca A., De Lucas-Consuegra A., Calcerrada A.B., Lobato J., Valverde J.L., Dorado F. (2013). From Biomass to Pure Hydrogen: Electrochemical Reforming of Bio-Ethanol in a PEM Electrolyser. Appl. Catal. B Environ..

[5] Holladay J.D., Hu J., King D.L., Wang Y. (2009). An Overview of Hydrogen Production Technologies. Catal. Today.

[6] Balaji R., Kannan B.S., Lakshmi J., Senthil N., Vasudevan S., Sozhan G., Shukla A.K., Ravichandran S. (2009). An Alternative Approach to Selective Sea Water Oxidation for Hydrogen Production. Electrochem. Commun..

[7] Gorensek M.B., Staser J.A., Stanford T.G., Weidner J.W. (2009). A Thermodynamic Analysis of the SO_2_/H_2_SO_4_ system in SO_2_-Depolarized Electrolysis. Int. J. Hydrogen Energy.

[8] Díaz-Abad S., Millán M., Rodrigo M.A., Lobato J. (2019). Review of Anodic Catalysts for SO_2_ Depolarized Electrolysis for “Green Hydrogen” Production. Catalysts.

[9] Lobato J., Díaz-Abad S., Peláez M.C., Millán M., Rodrigo M.A. (2019). Synthesis and Characterization of Pt on Novel Catalyst Supports for the H_2_ Production in the Westinghouse Cycle. Int. J. Hydrogen Energy.

[10] Fouzai I., Radaoui M., Díaz-Abad S., Rodrigo M.A., Lobato J. (2022). Electrospray Deposition of Catalyst Layers with Ultralow Pt Loading for Cost-Effective H_2_ Production by SO_2_ Electrolysis. ACS Appl. Energy Mater..

[11] Colón-Mercado H.R., Hobbs D.T. (2007). Catalyst Evaluation for a Sulfur Dioxide-Depolarized Electrolyzer. Electrochem. Commun..

[12] Meekins B.H., Thompson A.B., Gopal V., Mehrabadi B.A.T., Elvington M.C., Ganesan P., Newhouse-Illige T.A., Shepard A.W., Scipioni L.E., Greer J.A. (2020). In-Situ and Ex-Situ Comparison of the Electrochemical Oxidation of SO_2_ on Carbon Supported Pt and Au Catalysts. Int. J. Hydrogen Energy.

[13] Ding X., Chen S., Xiao P., Wang L., Zhang P. (2022). SO_2_-Depolarized Electrolysis Using Porous Graphite Felt as Diffusion Layer in Proton Exchange Membrane Electrolyzer. Int. J. Hydrogen Energy.

[14] Krüger A.J., Krieg H.M., Van Der Merwe J., Bessarabov D. (2014). Evaluation of MEA Manufacturing Parameters Using EIS for SO_2_ electrolysis. Int. J. Hydrogen Energy.

[15] Steimke J.L., Steeper T.J., Cólon-Mercado H.R., Gorensek M.B. (2015). Development and Testing of a PEM SO_2_-Depolarized Electrolyzer and an Operating Method That Prevents Sulfur Accumulation. Int. J. Hydrogen Energy.

[16] Staser J., Ramasamy R.P., Sivasubramanian P., Weidner J.W. (2007). Effect of Water on the Electrochemical Oxidation of Gas-Phase SO_2_ in a PEM Electrolyzer for H_2_ Production. Electrochem. Solid-State Lett..

[17] Ying Z., Yang J., Zheng X., Dou B., Cui G. (2022). Modeling and Numerical Study of SO_2_-Depolarized Electrolysis for Hydrogen Production in the Hybrid Sulfur Cycle. J. Clean. Prod..

[18] Gorensek M.B., Corgnale C., Summers W.A. (2017). Development of the Hybrid Sulfur Cycle for Use with Concentrated Solar Heat. I. Conceptual Design. Int. J. Hydrogen Energy.

[19] Gorensek M.B., Meekins B., Colón-Mercado H., Weidner J. (2020). Parametric Study of Operating Conditions of an SO_2_-Depolarized Electrolyzer. Int. J. Hydrogen Energy.

[20] Jayakumar J.V., Gulledge A., Staser J.A., Kim C.-H., Benicewicz B.C., Weidner J.W. (2012). Polybenzimidazole Membranes for Hydrogen and Sulfuric Acid Production in the Hybrid Sulfur Electrolyzer. ECS Electrochem. Lett..

[21] Peach R., Krieg H.M., Krüger A.J., Bessarabov D., Kerres J. (2018). PBI-Blended Membrane Evaluated in High Temperature SO_2_ Electrolyzer. ECS Trans..

[22] Díaz-Abad S., Rodrigo M.A., Lobato J. (2021). First Approaches for Hydrogen Production by the Depolarized Electrolysis of SO_2_ Using Phosphoric Acid Doped Polybenzimidazole Membranes. Int. J. Hydrogen Energy.

[23] Sun C., Negro E., Nale A., Pagot G., Vezzù K., Zawodzinski T.A., Meda L., Gambaro C., Di Noto V. (2021). An Efficient Barrier toward Vanadium Crossover in Redox Flow Batteries: The Bilayer [Nafion/(WO_3_)x] Hybrid Inorganic-Organic Membrane. Electrochim. Acta.

[24] Wong C.Y., Wong W.Y., Ramya K., Khalid M., Loh K.S., Daud W.R.W., Lim K.L., Walvekar R., Kadhum A.A.H. (2019). Additives in Proton Exchange Membranes for Low- and High-Temperature Fuel Cell Applications: A Review. Int. J. Hydrogen Energy.

[25] Artimani J.S., Ardjmand M., Enhessari M., Javanbakht M. (2020). High-Temperature PEMs Based on Polybenzimidazole and New Nanoparticles for Fuel Cell Application. J. Polym. Res..

[26] Üregen N., Pehlivanoğlu K., Özdemir Y., Devrim Y. (2017). Development of Polybenzimidazole/Graphene Oxide Composite Membranes for High Temperature PEM Fuel Cells. Int. J. Hydrogen Energy.

[27] Venkatkarthick R., Elamathi S., Sangeetha D., Balaji R., Suresh Kannan B., Vasudevan S., Jonas Davidson D., Sozhan G., Ravichandran S. (2013). Studies on Polymer Modified Metal Oxide Anode for Oxygen Evolution Reaction in Saline Water. J. Electroanal. Chem..

[28] Diaz-abad S., Fernández-Mancebo S., Rodrigo M.A., Lobato J. (2022). Characterization of PBI/Graphene Oxide Composite Membranes for the SO_2_ Depolarized Electrolysis at High Temperature. Membranes.

[29] Díaz-Abad S., Fernández-Mancebo S., Rodrigo M.A., Lobato J. (2022). Enhancement of SO_2_ High Temperature Depolarized Electrolysis by Means of Graphene Oxide Composite Polybenzimidazole Membranes. J. Clean. Prod..

[30] Lobato J., Cañizares P., Rodrigo M.A., Úbeda D., Pinar F.J. (2011). A Novel Titanium PBI-Based Composite Membrane for High Temperature PEMFCs. J. Memb. Sci..

[31] Pinar F.J., Cañizares P., Rodrigo M.A., Úbeda D., Lobato J. (2015). Long-Term Testing of a High-Temperature Proton Exchange Membrane Fuel Cell Short Stack Operated with Improved Polybenzimidazole-Based Composite Membranes. J. Power Sources.

[32] Kumar K.S., Prabhu M.R. (2021). Enhancing Proton Conduction of Poly(Benzimidazole) with Sulfonated Titania Nano Composite Membrane for PEM Fuel Cell Applications. Macromol. Res..

[33] Covaliu-Mierlă C.I., Matei E., Stoian O., Covaliu L., Constandache A.C., Iovu H., Paraschiv G. (2022). TiO_2_–Based Nanofibrous Membranes for Environmental Protection. Membranes.

[34] Lobato J., Cañizares P., Rodrigo M.A., Linares J.J., Manjavacas G. (2006). Synthesis and Characterisation of Poly [2,2-(m-Phenylene)-5,5-Bibenzimidazole] as Polymer Electrolyte Membrane for High Temperature PEMFCs. J. Memb. Sci..

[35] Lobato J., Cañizares P., Rodrigo M.A., Linares J.J., Aguilar J.A. (2007). Improved Polybenzimidazole Films for H_3_PO_4_-Doped PBI-Based High Temperature PEMFC. J. Memb. Sci..

[36] Davis J.R., Baygents J.C., Farrell J. (2014). Effect of Current Density and Sulfuric Acid Concentration on Persulfuric Acid Generation by Boron-Doped Diamond Film Anodes. J. Appl. Electrochem..

[37] Giannakis S., Lin K.Y.A., Ghanbari F. (2021). A Review of the Recent Advances on the Treatment of Industrial Wastewaters by Sulfate Radical-Based Advanced Oxidation Processes (SR-AOPs). Chem. Eng. J..

[38] Opperman H., Kerres J., Krieg H. (2012). SO_2_ Crossover Flux of Nafion^®^ and SFS-PBI Membranes Using a Chronocoulometric (CC) Monitoring Technique. J. Memb. Sci..

[39] Ijadpanah-Saravy H., Safari M., Khodadadi-Darban A., Rezaei A. (2014). Synthesis of Titanium Dioxide Nanoparticles for Photocatalytic Degradation of Cyanide in Wastewater. Anal. Lett..

[40] Kerres J., Atanasov V. (2015). Cross-Linked PBI-Based High-Temperature Membranes: Stability, Conductivity and Fuel Cell Performance. Int. J. Hydrogen Energy.

[41] Jian-hua T., Peng-fei G., Zhi-yuan Z., Wen-hui L., Zhong-qiang S. (2008). Preparation and Performance Evaluation of a Nafion-TiO_2_ Composite Membrane for PEMFCs. Int. J. Hydrogen Energy.

[42] Borup R., Meyers J., Pivovar B., Kim Y.S., Mukundan R., Garland N., Myers D., Wilson M., Garzon F., Wood D. (2007). Scientific Aspects of Polymer Electrolyte Fuel Cell Durability and Degradation. Chem. Rev..

[43] Mustarelli P., Quartarone E., Grandi S., Carollo A., Magistris A. (2008). Polybenzimidazole-Based Membranes as a Real Alternative to Nafion for Fuel Cells Operating at Low Temperature. Adv. Mater..

[44] Yu S., Xiao L., Benicewicz B.C. (2008). Durability Studies of PBI-Based High Temperature PEMFCs. Fuel Cells.

[45] Pinar F.J., Cañizares P., Rodrigo M.A., Ubeda D., Lobato J. (2012). Titanium Composite PBI-Based Membranes for High Temperature Polymer Electrolyte Membrane Fuel Cells. Effect on Titanium Dioxide Amount. RSC Adv..

[46] Ma Y.-L., Wainright J.S., Litt M.H., Savinell R.F. (2004). Conductivity of PBI Membranes for High-Temperature Polymer Electrolyte Fuel Cells. J. Electrochem. Soc..

[47] Santasalo-Aarnio A., Virtanen J., Gasik M. (2016). SO_2_ Carry-over and Sulphur Formation in a SO_2_-Depolarized Electrolyser. J. Solid State Electrochem..

[48] Santasalo-Aarnio A., Lokkiluoto A., Virtanen J., Gasik M.M. (2016). Performance of Electrocatalytic Gold Coating on Bipolar Plates for SO_2_depolarized Electrolyser. J. Power Sources.

[49] Allen J.A., Rowe G., Hinkley J.T., Donne S.W. (2014). Electrochemical Aspects of the Hybrid Sulfur Cycle for Large Scale Hydrogen Production. Int. J. Hydrogen Energy.

