# Supplementary material for: Enhancement of the Green H2 Production by Using TiO2 Composite Polybenzimidazole Membranes
Source: Nanomaterials (Basel). 2022 Aug 24;12(17):2920. doi: 10.3390/nano12172920 (PMC9457720; doi:10.3390/nano12172920)
Supplement: Supplementary file 1 [file nanomaterials-12-02920-s001.zip › nanomaterials-1829822-supplementary.pdf]

nanomaterials

MDPI

Supplementary Materials

Enhancement of the Green H_2 Production by Using TiO_2 Composite Polybenzimidazole Membranes

Sergio Díaz-Abad, Manuel A. Rodrigo, Cristina Sáez and Justo Lobato *

Chemical Engineering Department, Enrique Costa Building, University of Castilla-La Mancha, Av. Camilo Jose Cela n 12, 13071 Ciudad Real, Spain

*	Correspondence: justo.lobato@uclm.es

Citation: Díaz-Abad, S.; Rodrigo, M.A.; Sáez, C.; Lobato, J. Enhancement of the Green H_2 Production by Using TiO_2 Composite Polybenzimidazole Membranes. *Nanomaterials* **2022**, *12*, 2920. https://doi.org/10.3390/nano12172920

Academic Editor: Filipe M.L. Figueiredo

Received: 7 July 2022
Accepted: 22 August 2022
Published: 24 August 2022

Publisher's Note: MDPI stays neutral with regard to jurisdictional claims in published maps and institutional affiliations.

Copyright: © 2022 by the authors. Submitted for possible open access publication under the terms and conditions of the Creative Commons Attribution (CC BY) license (https://creativecommons.org/licenses/by/4.0/).

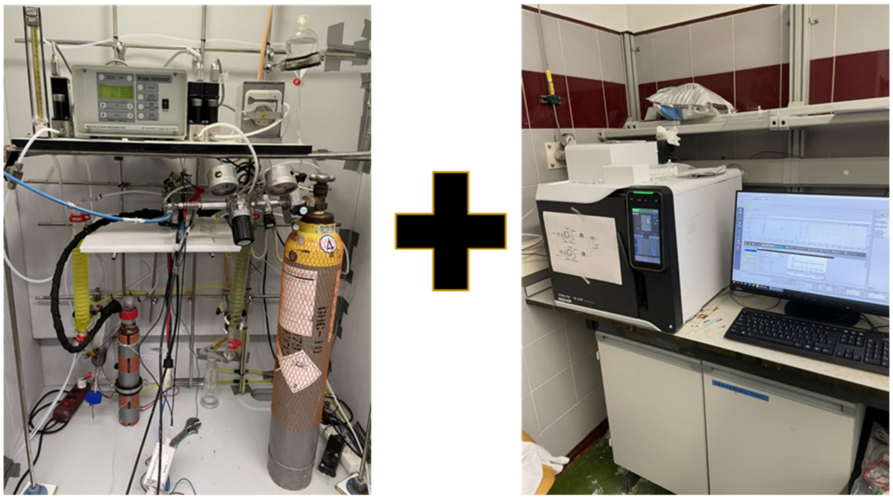

Figure S1. Experimental set-up.

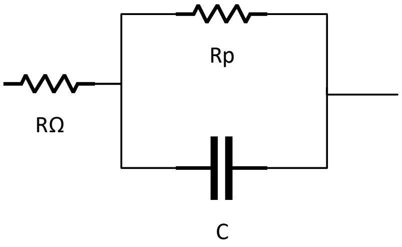

Figure S2. (RC) circuit use to fit the EIS data with the software NOVA.

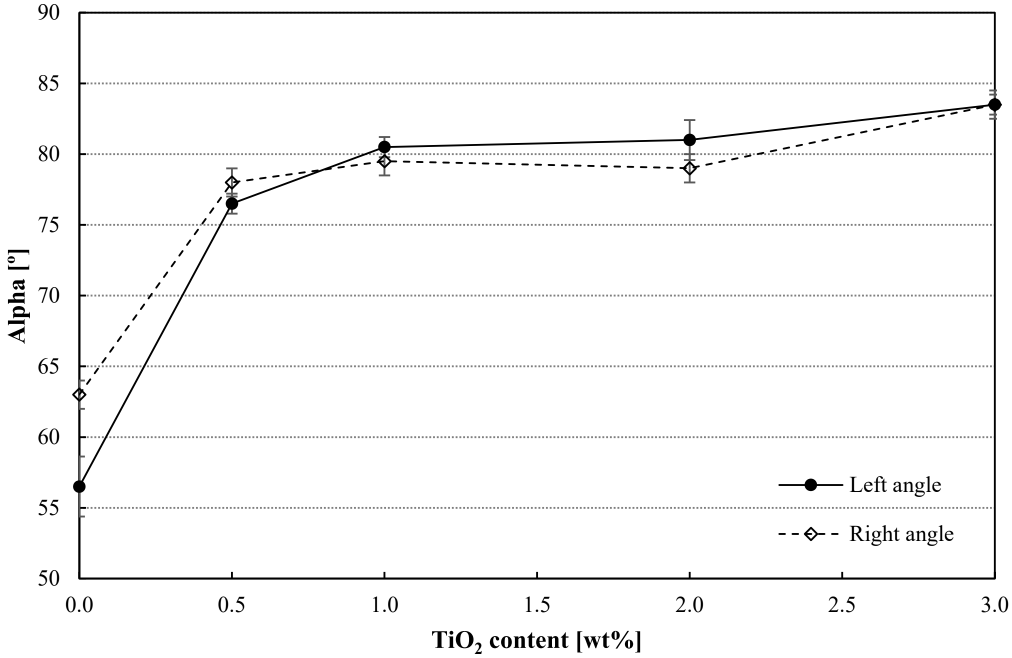

Figure S3. Contact angle for the different TiO_2 concentrations.

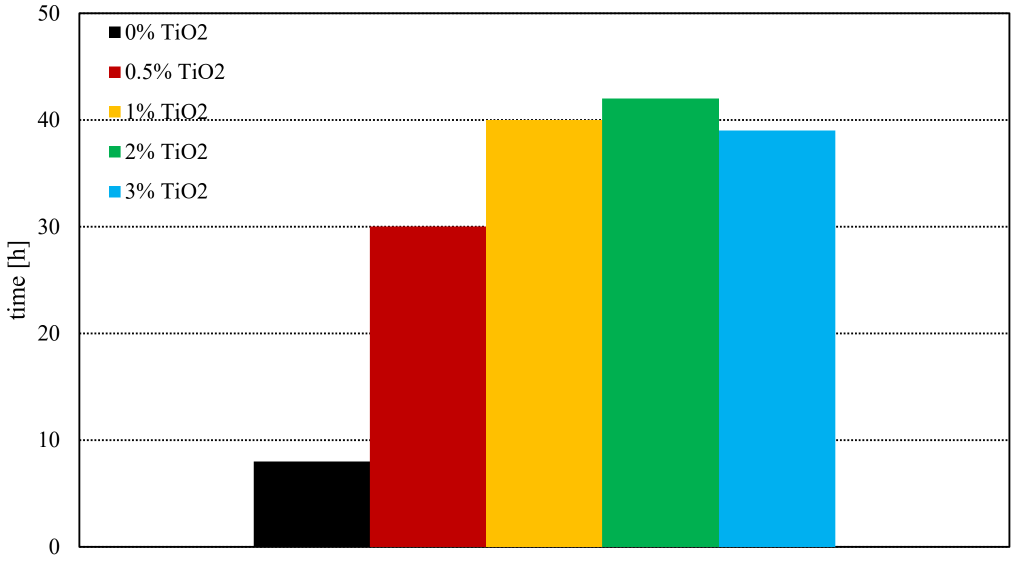

Figure S4. Chemical stability test. Time at which each membrane failed.

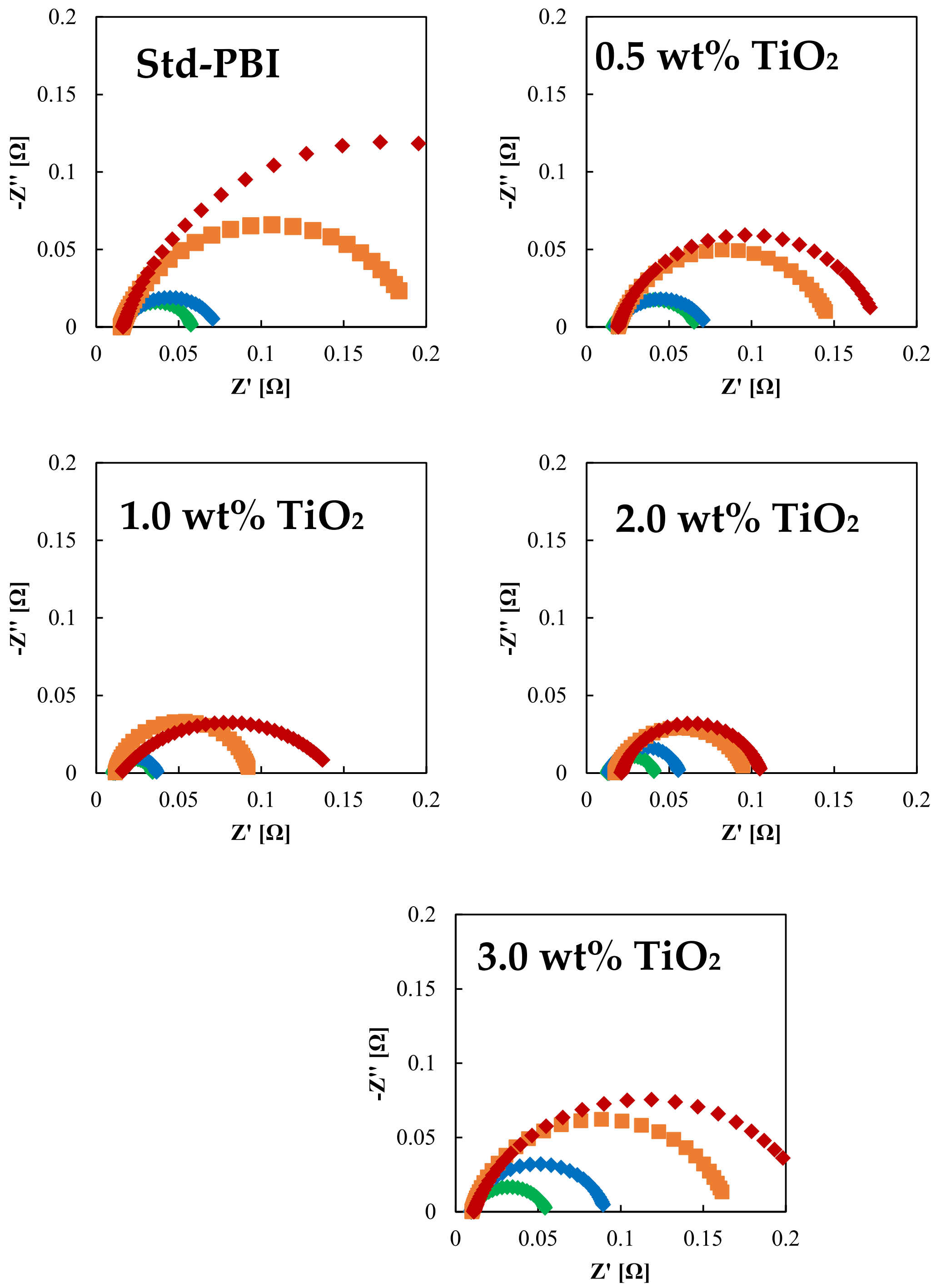

Figure S5. Nyquist plots at 0.7 V for the studied membranes. Green dots: 110 °C; Blue dots: 120 °C; Orange dots: 130 °C; Red dots: 140 °C.

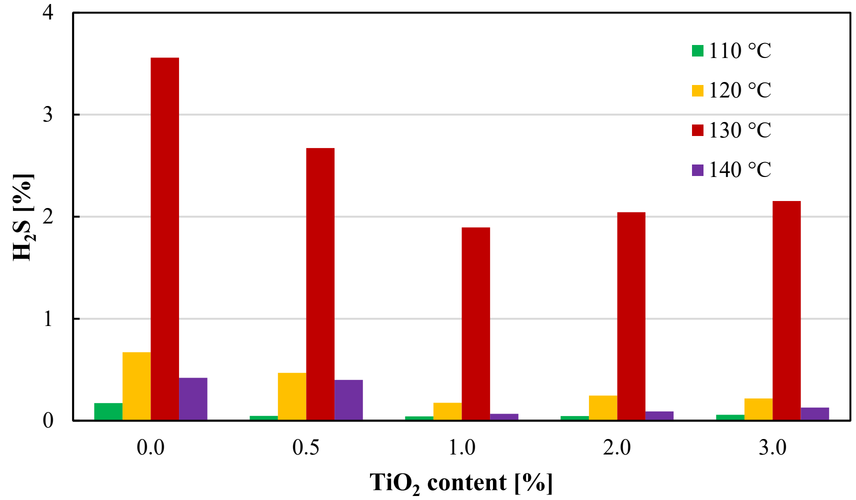

Figure S6. H_2S contents in the cathode outlet measured at 0.6 V.

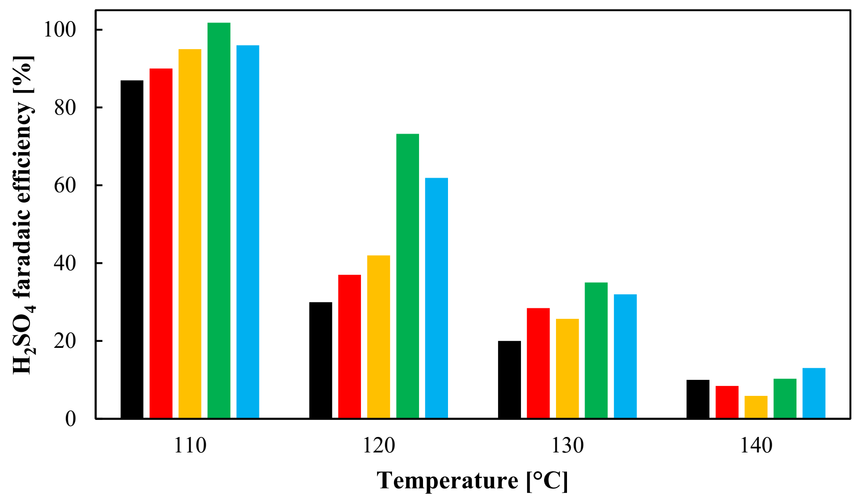

Figure S7. H_2SO_4 production efficiency in the anode measured at 0.6 V. Black column: Std-PBI; Red column: 0.5 wt% TiO_2-PBI; Yellow column: 1.0 wt% TiO_2-PBI; Green column: 2.0 wt% TiO_2-PBI; Blue column: 3.0 wt% TiO_2-PBI.

Table S1. SO_2 crossover flux.

**Membrane**	**Crossover flux [mol SO_2 cm^{-2} s^{-1} · 10^{-5}]**			
	**110 °C**	**120 °C**	**130 °C**	**140 °C**
Std-PBI	3.40	3.56	4.23	4.50
0.5 wt% TiO_2-PBI	3.07	3.15	3.73	4.21
1.0 wt% TiO_2-PBI	2.32	2.57	3.40	3.98
2.0 wt% TiO_2-PBI	1.74	1.99	3.07	3.80
3.0 wt% TiO_2-PBI	1.53	1.70	2.90	3.66
